# MeSS and assembly_finder: a toolkit for *in silico* metagenomic sample generation

**DOI:** 10.1093/bioinformatics/btae760

**Published:** 2024-12-31

**Authors:** Farid Chaabane, Trestan Pillonel, Claire Bertelli

**Affiliations:** Institute of Microbiology, Lausanne University Hospital and University of Lausanne, Lausanne, 1011, Switzerland; Institute of Microbiology, Lausanne University Hospital and University of Lausanne, Lausanne, 1011, Switzerland; Institute of Microbiology, Lausanne University Hospital and University of Lausanne, Lausanne, 1011, Switzerland

## Abstract

**Summary:**

The intrinsic complexity of the microbiota combined with technical variability render shotgun metagenomics challenging to analyze for routine clinical or research applications. *In silico* data generation offers a controlled environment allowing for example to benchmark bioinformatics tools, to optimize study design, statistical power, or to validate targeted applications. Here, we propose assembly_finder and the Metagenomic Sequence Simulator (MeSS), two easy-to-use Bioconda packages, as part of a benchmarking toolkit to download genomes and simulate shotgun metagenomics samples, respectively. Outperforming existing tools in speed while requiring less memory, MeSS reproducibly generates accurate complex communities based on a list of taxonomic ranks and their abundance.

**Availability and implementation:**

All code is released under MIT License and is available on https://github.com/metagenlab/MeSS and https://github.com/metagenlab/assembly_finder.

## 1 Introduction

Shotgun metagenomics has been widely applied for large-scale investigations of complex human, animal, and environmental microbiomes (Huttenhower *et al.* 2012, Oh *et al.* 2014, Quince *et al.* 2017). While species composition and functions correlated with various diseases (Zhernakova *et al.* 2016) or environmental conditions (Techtmann and Hazen 2016), detecting statistically significant biological effect sizes between the microbiome and outcomes is challenging (Quince *et al.* 2017) and must be backed by adequately powered studies. Similarly, the translation of metagenomics to routine clinical diagnosis requires extensive testing to validate each application and assess its limits (Scherz *et al.* 2022). However, validating shotgun metagenomics by sequencing real samples is expensive and burdensome (Afshinnekoo *et al.* 2017). Thus, data emulation is a cost- and time-effective approach for researchers and clinical microbiologists for example to assess workflow performance, study design, and power, or to benchmark bioinformatic tools (Gourlé *et al.* 2019).

Tools for microbiome simulation employ different strategies, including some using real data. For example, MB-GAN uses a generative adversarial network (Rong *et al.* 2021) and learns from existing microbiome abundances to simulate realistic abundances. Other tools such as SEQ2MGS (Van Camp and Porollo 2022) rely on real sequencing reads and mixes them together to generate artificial metagenomes. While using existing high-throughput sequencing data generates realistic benchmarking datasets, the data can contain errors, biases and uncertainties that can pose challenges for data interpretation (Pfeifer 2017). In addition, the impact of computational methods on results is huge as different tools provide divergent results in genomic analyses like variant calling (Barbitoff *et al.* 2022). Thus, a controlled environment, without experimental biases, is needed to correctly benchmark computational methods performance (Milhaven and Pfeifer 2023). Read simulators, which emulate synthetic DNA sequencing reads based on sequencing technologies specific error models, can be used to construct such an environment. Popular short read simulators (Milhaven and Pfeifer 2023) include ART (Huang *et al.* 2012), InSilicoSeq (Gourlé *et al.* 2019), Mason (Holtgrewe 2010), and NEAT (Stephens *et al.* 2016). For long read sequencing, tools like SILICO (Baker *et al.* 2016), Badread (Wick 2019), and PBSIM3 (Ono *et al.* 2022) allow both Nanopore and PacBio reads simulation. Most of the read simulators are used to generate whole genome sequencing reads for one genome. Thus, generating sequencing reads for multiple genomes with fine-grain control on abundance profiles for each genome is complicated. To our knowledge, only CAMISIM (Fritz *et al.* 2019) offers the possibility to generate *in silico* metagenome sequences of communities for both long and short reads technologies. In fact, CAMISIM simulates metagenomics sequences out of existing 16S profiles or *de novo*, from a list of genomes (Fritz *et al.* 2019). CAMISIM offers many customization options like multi-sample time series, but sample generation is slow and only few reference datasets are available on the CAMI challenge website (Meyer *et al.* 2022).

Here, we propose MeSS and assembly_finder, easy-to-use Bioconda (Grüning *et al.* 2018) packages that allow scalable and reproducible reference genome download as well as short and long read simulation to mimic shotgun metagenomics sequencing of complex samples.

## 2 Features

MeSS streamlines metagenomic sample generation by automating genome download, community design and read simulation in one command line. MeSS takes as input a table of genomes with their abundances, to produce shuffled, anonymous and compressed reads, and optionally their alignment to their reference sequence. Since reads and alignment files can occupy large volumes (Numanagić *et al.* 2016), they are compressed by default to minimize storage footprint (Sun *et al.* 2021). In addition, MeSS outputs taxonomic profiles, in biobox format, containing exact expected sequence and taxonomic abundances (Sun *et al.* 2021). To ensure scalability and efficient resource usage, users can set the number of CPUs and memory required by the pipeline and use built-in scheduler profiles like SLURM (Yoo *et al.* 2003) to run on high-performance computing clusters. Lastly, MeSS provides an easy subcommand to create metagenome templates for healthy individuals covering four human body sites. These templates can be used to create scenario-based simulations like spiking in a respiratory pathogen to mimic an infection of respiratory airways.

Read simulation involves running command line tools that require different software dependencies and file format processing, which makes it tedious for inexperienced users to run manually. To address this, MeSS uses Snakemake, a workflow engine that automates steps ensuring scalability and reproducibility while facilitating software dependency deployment (Köster and Rahmann 2012). Despite these advantages, running a Snakemake pipeline requires extensive setup, from copying the workflow directory, configuration file modification to inputting the absolute path to a Snakefile, which can be overwhelming for new users (Roach *et al.* 2022). To improve user experience, MeSS uses Snaketool (Roach *et al.* 2022) to conveniently launch Snakemake from the command line without the previously mentioned hassles.

### 2.1 Assembly download with the Bioconda package assembly_finder

**Figure 1.**
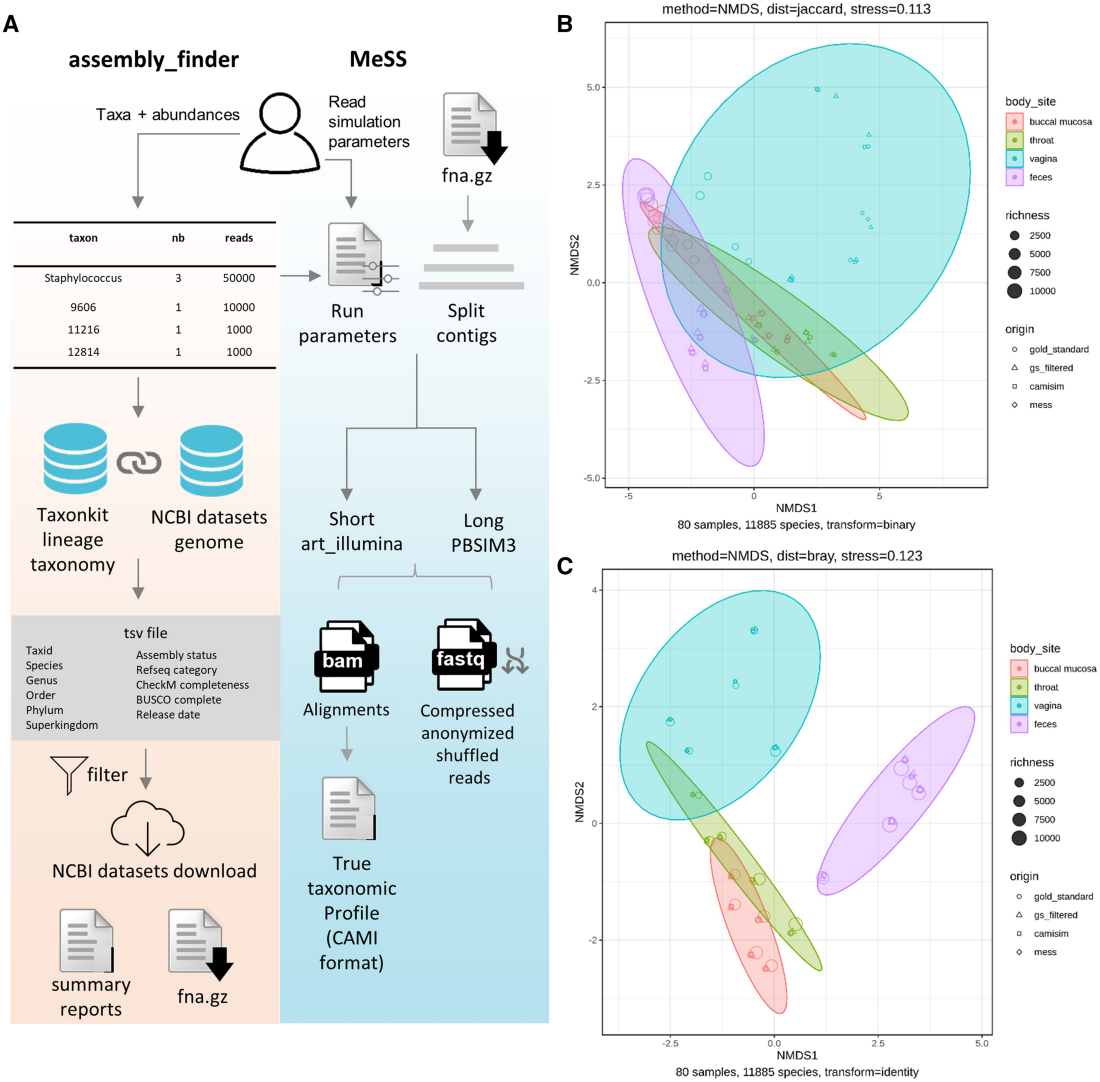
MeSS and assembly_finder workflows and assessment of simulated communities. (A) Main steps of assembly_finder and MeSS workflows. (B) Analysis of real and simulated datasets with NMDS ordination of beta diversity using Jaccard. (C) Bray–Curtis. Circles correspond to real samples, triangles to real samples filtered at 200 reads per taxa, squares and diamonds to CAMISIM and MeSS simulated samples, respectively. Shape size reflects the number of species present in the sample. Each ellipse groups a different body site: buccal mucosa (red), throat (green), vagina (blue), and feces (purple).

To simulate metagenomic sequences of microbial communities, genome assemblies of representative taxa can be downloaded from public databases such as NCBI RefSeq (O’Leary *et al.* 2016). While the assembly summary table in NCBI’s ftp contains all links to access the data, their download via ftp is slow and often results in connection timeouts. Therefore, we developed assembly_finder (Fig. 1A), a wrapper around the NCBI datasets command line (O’Leary *et al.* 2024) to retrieve large numbers of genome sequences. Starting from lists of scientific names, taxonomic identifiers (taxids) (Federhen 2012), or genome accessions, assembly_finder combines NCBI datasets commands, for a fast and easy download of genome sequences and metadata.

For each query, assembly metadata like assembly status, assembly level, and taxonomy identifiers are retrieved using the datasets summary genome command. For all unique taxonomy identifiers a complete lineage taxonomy, from kingdom to species is retrieved using taxonkit (Shen and Ren 2021). Next, genome assemblies are ranked according to metrics present in the assembly metadata table, which include RefSeq category (reference, or representative), assembly status (complete, chromosome, scaffold, contig), BUSCO (Manni *et al.* 2021a,b) and checkM (Parks *et al.* 2015) completeness scores. BUSCO and checkM completeness estimate genome quality based on expected gene content (Manni *et al.* 2021a,b), and thus, genomes with high BUSCO and checkM scores rank higher in the summary table. Optionally, assembly_finder can rank assemblies at any given taxonomic level, for example to download the 10 best-ranking genomes for each bacterial genus.

### 2.2 Sample community design

For each metagenomic sample, MeSS accepts a list of local genomes or a list of taxa to be downloaded with assembly_finder. By default, MeSS supports manually assigning quantities to each taxon or genome. For example, sequence counts (bases or reads), sequence abundances, or taxonomic abundances can be set and are converted to coverage depths by normalizing to genome sizes. Alternatively, MeSS can draw abundances from an even (same taxon with equal proportions) or a log-normal distribution, frequently used to model microbial communities (Curtis *et al.* 2002, Unterseher *et al.* 2011).

### 2.3 Read simulation

To choose read simulators to include in MeSS, we established the following selection criteria. First, the simulator should generate fastq files. Second, it must generate a “ground truth” SAM/BAM file to know the genomic location of reads, which is crucial for benchmarking and tool comparison (Milhaven and Pfeifer 2023). Third, it needs low computational costs and must be installable via bioconda. As both ART and PBSIM3 fit the criteria and ART performed well in benchmarks (Milhaven and Pfeifer 2023), they were included in MeSS for short and long read simulation, respectively. ART simulates an equal amount of reads per contig (Milhaven and Pfeifer 2023) and PBSIM3 generates reads per contig. Thus, to have a uniform read sampling across the genome for ART, and compatibility with PBSIM3, multi contig fasta files are split into separate contig files. Simulated reads per contig are then concatenated into one paired or single fastq file per sample. Simulated reads contain information like fasta header name, and genome position from which the read was sampled. To hide this information in the anonymized reads, read headers are replaced by their index position in the fastq file using seqkit (Shen *et al.* 2016). Moreover, to avoid downstream biases in the analysis, reads are shuffled with seqkit as well. Optionally, MeSS generates as output alignment files which are converted to the BAM format using SAMtools (Li *et al.* 2009) and Bioconvert (Caro *et al.* 2023). Average coverage and read counts are extracted from BAMs using “samtools coverage” (Li *et al.* 2009), which is then used to create a taxonomic profile file following the biobox format (Belmann *et al.* 2015) using “taxonkit profile2cami” (Shen and Ren 2021). As highlighted by Sun *et al.*, metagenomics profiles can contain either relative sequence or taxonomic abundances, and neglecting the difference between these two metrics can bias benchmarking results (Sun *et al.* 2021). To avoid this, MeSS provides profiles with both sequence (read percentages) and taxonomic (coverage depth percentages) abundances.

Technical replicates from the same metagenome can be generated by setting the number of replicates and the standard deviation of genome quantity value (reads, bases, coverage depths, and sequence or taxonomic abundances) between replicates. By default, the standard deviation between replicates is zero, and the difference is the sequence genomic origin set by the seed. Overall, the pipeline outputs compressed fastq files, a true taxonomic abundance profile in CAMI format, as well as BAM files to verify reads alignments to their reference genome.

## 3 Benchmarks

### 3.1 Comparison of simulated and real samples

To evaluate MeSS ability to generate realistic metagenomes, 20 samples from four different body sites (buccal mucosa, throat, gut, and vagina) were retrieved from the Human Microbiome Project (Huttenhower *et al.* 2012). Reads were classified with Kraken2 (Wood *et al.* 2019) using a standard Kraken2 index from June 2024 obtained from Index zone. Sequence abundances were estimated at the species level using Bracken (Lu *et al.* 2017). Across all 20 samples, 1498 unique taxa, to which at least 200 reads were attributed at species level, formed the real expected community. Species taxids were inputted to assembly_finder to download one reference genome per taxid. The downloaded genomes were used for read simulation using MeSS and CAMISIM, the current state-of-the-art metagenomic sample simulator (Fritz *et al.* 2019). To ensure a fair comparison, we ran MeSS and CAMISIM with the same parameters to simulate 100 bp paired reads following the empHiseq2k error profile using 16 CPUs within a Nextflow (Di Tommaso *et al.* 2017) pipeline available here (https://github.com/metagenlab/benchmark-MeSS-CAMISIM). To demonstrate that simulated samples have the same community structure as real samples, we evaluated alpha and beta diversity indexes. As shown in Supplementary Fig. S1, all alpha diversity indices evaluating richness and/or abundance from simulated and real datasets strongly correlate (0.96 Spearman’s R squared, on average, across all indexes). This demonstrates that MeSS simulates mock communities with similar evenness and richness compared to expected communities.

To assess whether species composition and abundance in simulated samples are comparable to the real communities, pairwise Jaccard and Bray–Curtis distances were computed and visualized by NMDS ordination plots (Fig. 1B). Real samples cluster closely with their simulated counterparts, and group by body sites, with some expected similarities between throat and mouth metagenomes. A PERMANOVA with Bray–Curtis distance showed that centroids and dispersion differ significantly among body sites (*F* = 16.05, *P* = 0.001), but not among simulated and real communities (*F* = 0.08, *P* = 1.000), confirming that the species and their relative abundances are conserved between real and simulated sample metagenomes. The code to reproduce the figures is available at https://github.com/metagenlab/MeSS-figures.

### 3.2 Resource usage

CPU and RAM usage of MeSS was compared with CAMISIM *de novo* mode using 2000 bacterial reference genomes. Sixteen subsamples were simulated with an increasing number of genomes, first ranging from 1 to 10, and then doubling up to 640, and a coverage depth of 1x. As shown in Supplementary Fig. S2, MeSS exhibits a lower linear time complexity when compared to CAMISIM. Time complexity is defined as the process time needed to simulate metagenomes as a function of the number of genomes. Overall, MeSS is around 18x faster than CAMISIM and uses less RAM (Supplementary Fig. S3). The code to benchmark resources usage is available at https://github.com/metagenlab/benchmark-MeSS-CAMISIM.

## 4 Conclusion

assembly_finder together with MeSS can be used as a scalable, reproducible, and easy-to-use toolkit for benchmarking metagenomic software. In fact, with its simple command line, one can gather genomes and mix them into communities to mimic shotgun metagenomics sequencing of complex communities. It offers fast assembly download and parallel read simulation with an extensive list of parameters such as number of replicates, choice of sequencing technology, sequencing error profile, and read pairing. MeSS can generate specific communities, either inferred from taxonomic profiling of real metagenomic samples, or communities with random lognormal or even distributions. Moreover, we bundled metagenome templates for healthy individuals, which can be useful to create scenario-based simulations for example by spiking in microorganisms to benchmark tools for pathogen detection. MeSS offers improved efficiency to available computational resources, outperforming CAMISIM. Both MeSS and assembly_finder source codes are available at https://github.com/metagenlab/MeSS and https://github.com/metagenlab/assembly_finder.

## Supplementary Material

btae760_Supplementary_Data

## References

[btae760-B1] Afshinnekoo E , Chou C, Alexander N et al Precision metagenomics: rapid metagenomic analyses for infectious disease diagnostics and public health surveillance. J Biomol Tech 2017;28:40–5. 28337072 10.7171/jbt.17-2801-007 PMC5360386

[btae760-B2] Baker EAG, Goodwin S, McCombie R et al *SiLiCO: A Simulator of Long Read Sequencing in PacBio and Oxford Nanopore*. bioRxiv, 2016.

[btae760-B3] Barbitoff YA , Abasov R, Tvorogova VE et al Systematic benchmark of state-of-the-art variant calling pipelines identifies major factors affecting accuracy of coding sequence variant discovery. BMC Genomics 2022;23:155. 35193511 10.1186/s12864-022-08365-3 PMC8862519

[btae760-B4] Belmann P , Dröge J, Bremges A et al Bioboxes: standardised containers for interchangeable bioinformatics software. Gigascience 2015;4:47–0. 26473029 10.1186/s13742-015-0087-0 PMC4607242

[btae760-B5] Caro H , Dollin S, Biton A et al BioConvert: a comprehensive format converter for life sciences. NAR Genom Bioinform 2023;5:lqad074. 10.1093/nargab/lqad074 PMC10440784 37608802

[btae760-B6] Curtis TP , Sloan WT, Scannell JW et al Estimating prokaryotic diversity and its limits. Proc Natl Acad Sci USA 2002;99:10494–9. 12097644 10.1073/pnas.142680199 PMC124953

[btae760-B7] Di Tommaso P , Chatzou M, Floden EW et al Nextflow enables reproducible computational workflows. Nat Biotechnol 2017;35:316–9. 28398311 10.1038/nbt.3820

[btae760-B8] Federhen S. The NCBI taxonomy database. Nucleic Acids Res 2012;40:D136–43. 22139910 10.1093/nar/gkr1178 PMC3245000

[btae760-B9] Fritz A , Hofmann P, Majda S et al CAMISIM: simulating metagenomes and microbial communities. Microbiome 2019;7:17. 30736849 10.1186/s40168-019-0633-6 PMC6368784

[btae760-B10] Gourlé H , Karlsson-Lindsjö O, Hayer J et al Simulating Illumina metagenomic data with InSilicoSeq. Bioinformatics 2019;35:521–2. 30016412 10.1093/bioinformatics/bty630 PMC6361232

[btae760-B11] Grüning B , Dale R, Sjödin A et al; Bioconda Team. Bioconda: sustainable and comprehensive software distribution for the life sciences. Nat Methods 2018;15:475–6. 29967506 10.1038/s41592-018-0046-7 PMC11070151

[btae760-B12] Holtgrewe M. Mason—A Read Simulator for Second Generation Sequencing Data. Technical Report FU Berlin, 2010.

[btae760-B13] Huang W , Li L, Myers JR et al ART: a next-generation sequencing read simulator. Bioinformatics 2012;28:593–4. 22199392 10.1093/bioinformatics/btr708 PMC3278762

[btae760-B14] Huttenhower C, Gevers D, Knigh R et al Structure, function and diversity of the healthy human microbiome. Nature 2012;486:207–14. 22699609 10.1038/nature11234 PMC3564958

[btae760-B15] Köster J , Rahmann S. Snakemake—a scalable bioinformatics workflow engine. Bioinformatics 2012;28:2520–2. 22908215 10.1093/bioinformatics/bts480

[btae760-B16] Li H , Handsaker B, Wysoker A et al; 1000 Genome Project Data Processing Subgroup. The sequence alignment/map format and SAMtools. Bioinformatics 2009;25:2078–9. 19505943 10.1093/bioinformatics/btp352 PMC2723002

[btae760-B17] Lu J , Breitwieser FP, Thielen P et al Bracken: estimating species abundance in metagenomics data. PeerJ Comput Sci 2017;3:e104. 10.7717/peerj-cs.104 PMC12016282 40271438

[btae760-B18] Manni M , Berkeley MR, Seppey M et al BUSCO: assessing genomic data quality and Beyond. Curr Protoc2021a;1:e323. 34936221 10.1002/cpz1.323

[btae760-B19] Manni M , Berkeley MR, Seppey M et al BUSCO update: novel and streamlined workflows along with broader and deeper phylogenetic coverage for scoring of eukaryotic, prokaryotic, and viral genomes. Mol Biol Evol2021b;38:4647–54. 34320186 10.1093/molbev/msab199 PMC8476166

[btae760-B20] Meyer F , Fritz A, Deng Z-L et al Critical assessment of metagenome interpretation: the second round of challenges. Nat Methods 2022;19:429–40. 35396482 10.1038/s41592-022-01431-4 PMC9007738

[btae760-B21] Milhaven M , Pfeifer SP. Performance evaluation of six popular short-read simulators. Heredity (Edinb) 2023;130:55–63. 36496447 10.1038/s41437-022-00577-3 PMC9905089

[btae760-B22] Numanagić I , Bonfield JK, Hach F et al Comparison of high-throughput sequencing data compression tools. Nat Methods 2016;13:1005–8. 27776113 10.1038/nmeth.4037

[btae760-B23] Oh J , Byrd AL, Deming C et al; NISC Comparative Sequencing Program. Biogeography and individuality shape function in the human skin metagenome. Nature 2014;514:59–64. 25279917 10.1038/nature13786 PMC4185404

[btae760-B24] O’Leary NA , Cox E, Holmes JB et al Exploring and retrieving sequence and metadata for species across the tree of life with NCBI datasets. Sci Data 2024;11:732. 38969627 10.1038/s41597-024-03571-y PMC11226681

[btae760-B25] O’Leary NA, Wright MW, Brister JR et al Reference sequence (RefSeq) database at NCBI: current status, taxonomic expansion, and functional annotation. Nucleic Acids Res 2016;44:D733–45. 26553804 10.1093/nar/gkv1189 PMC4702849

[btae760-B26] Ono Y , Hamada M, Asai K et al PBSIM3: a simulator for all types of PacBio and ONT long reads. NAR Genom Bioinform 2022;4:lqac092. 36465498 10.1093/nargab/lqac092 PMC9713900

[btae760-B27] Parks DH , Imelfort M, Skennerton CT et al CheckM: assessing the quality of microbial genomes recovered from isolates, single cells, and metagenomes. Genome Res 2015;25:1043–55. 25977477 10.1101/gr.186072.114 PMC4484387

[btae760-B28] Pfeifer SP. From next-generation resequencing reads to a high-quality variant data set. Heredity 2017;118:111–24. 27759079 10.1038/hdy.2016.102 PMC5234474

[btae760-B29] Quince C , Walker AW, Simpson JT et al Shotgun metagenomics, from sampling to analysis. Nat Biotechnol 2017;35:833–44. 28898207 10.1038/nbt.3935

[btae760-B30] Roach MJ , Pierce-Ward NT, Suchecki R et al Ten simple rules and a template for creating workflows-as-applications. PLoS Comput Biol 2022;18:e1010705. 36520686 10.1371/journal.pcbi.1010705 PMC9754251

[btae760-B31] Rong R , Jiang S, Xu L et al MB-GAN: microbiome simulation via generative adversarial network. Gigascience 2021;10:giab005. 33543271 10.1093/gigascience/giab005 PMC7931821

[btae760-B32] Scherz V , Greub G, Bertelli C et al Building up a clinical microbiota profiling: a quality framework proposal. Crit Rev Microbiol 2022;48:356–75. 34752719 10.1080/1040841X.2021.1975642

[btae760-B33] Shen W , Le S, Li Y et al SeqKit: a cross-platform and ultrafast toolkit for FASTA/Q file manipulation. PLoS One 2016;11:e0163962. 27706213 10.1371/journal.pone.0163962 PMC5051824

[btae760-B34] Shen W , Ren H. TaxonKit: a practical and efficient NCBI taxonomy toolkit. J Genet Genomics 2021;48:844–50. 34001434 10.1016/j.jgg.2021.03.006

[btae760-B35] Stephens ZD , Hudson ME, Mainzer LS et al Simulating next-generation sequencing datasets from empirical mutation and sequencing models. PLoS One 2016;11:e0167047. 27893777 10.1371/journal.pone.0167047 PMC5125660

[btae760-B36] Sun Z , Huang S, Zhang M et al Challenges in benchmarking metagenomic profilers. Nat Methods 2021;18:618–26. 33986544 10.1038/s41592-021-01141-3 PMC8184642

[btae760-B37] Techtmann SM , Hazen TC. Metagenomic applications in environmental monitoring and bioremediation. J Ind Microbiol Biotechnol 2016;43:1345–54. 27558781 10.1007/s10295-016-1809-8

[btae760-B38] Unterseher M , Jumpponen A, Opik M et al Species abundance distributions and richness estimations in fungal metagenomics—lessons learned from community ecology. Mol Ecol 2011;20:275–85. 21155911 10.1111/j.1365-294X.2010.04948.x

[btae760-B39] Van Camp J , Porollo A. SEQ2MGS: an effective tool for generating realistic artificial metagenomes from the existing sequencing data. NAR Genom Bioinform 2022;4:lqac050. 35899079 10.1093/nargab/lqac050 PMC9310082

[btae760-B40] Wick RR. Badread: simulation of error-prone long reads. J Open Source Softw 2019;4:1316.

[btae760-B41] Wood DE , Lu J, Langmead B et al Improved metagenomic analysis with Kraken 2. Genome Biol 2019;20:257. 31779668 10.1186/s13059-019-1891-0 PMC6883579

[btae760-B42] Yoo AB , Jette MA, Grondona M. SLURM: simple linux utility for resource management. In: Feitelson D et al (eds.), Job Scheduling Strategies for Parallel Processing. Berlin, Heidelberg: Springer, 2003, 44–60.

[btae760-B43] Zhernakova A , Kurilshikov A, Bonder MJ et al; LifeLines Cohort Study. Population-based metagenomics analysis reveals markers for gut microbiome composition and diversity. Science 2016;352:565–9. 27126040 10.1126/science.aad3369 PMC5240844

