# Supplementary material for: MeSS and assembly_finder: a toolkit for in silico metagenomic sample generation
Source: Bioinformatics. 2024 Dec 31;41(1):btae760. doi: 10.1093/bioinformatics/btae760 (PMC11755095; doi:10.1093/bioinformatics/btae760)
Supplement: btae760_Supplementary_Data [file btae760_supplementary_data.zip › 3576f_Chaabane_MeSS_sup_material.docx]

**MeSS and assembly_finder: A toolkit for *in silico* metagenomic sample generation**

Farid Chaabane, Trestan Pillonel, Claire Bertelli

*Institute of Microbiology, Lausanne University Hospital and University of Lausanne, Lausanne, Switzerland*

**Corresponding author:** Claire Bertelli


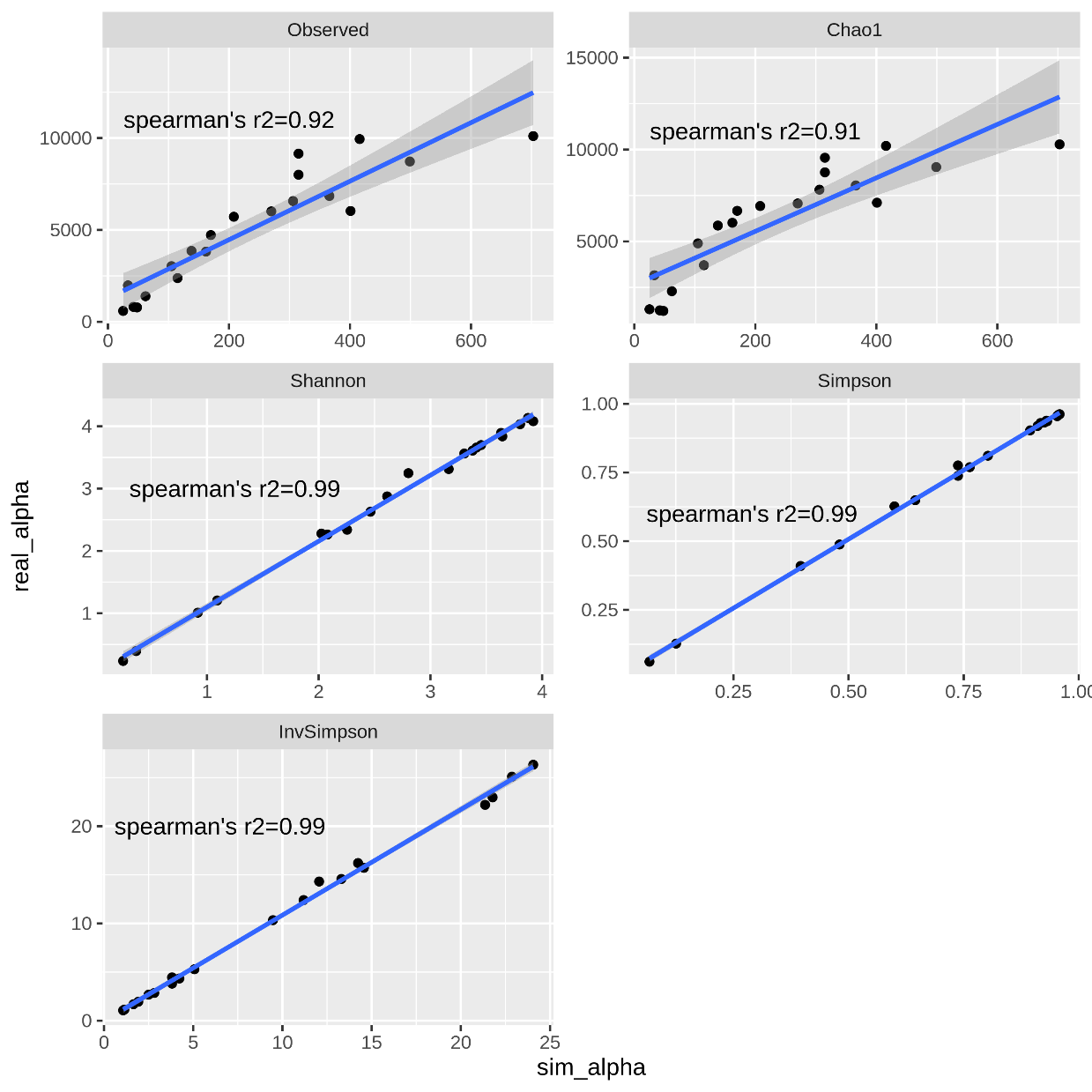
All code for raw data generation and figures are under the [MeSS-figures](https://github.com/metagenlab/MeSS-figures) repository.

***Figure S1: Correlation between alpha diversity inferred from simulated and real datasets.*** *The alpha diversity of simulated (x axis) and real (y axis) shotgun metagenomics samples was measured by observed, Chao1, Shannon, and Inverse Simpson diversity indexes.*


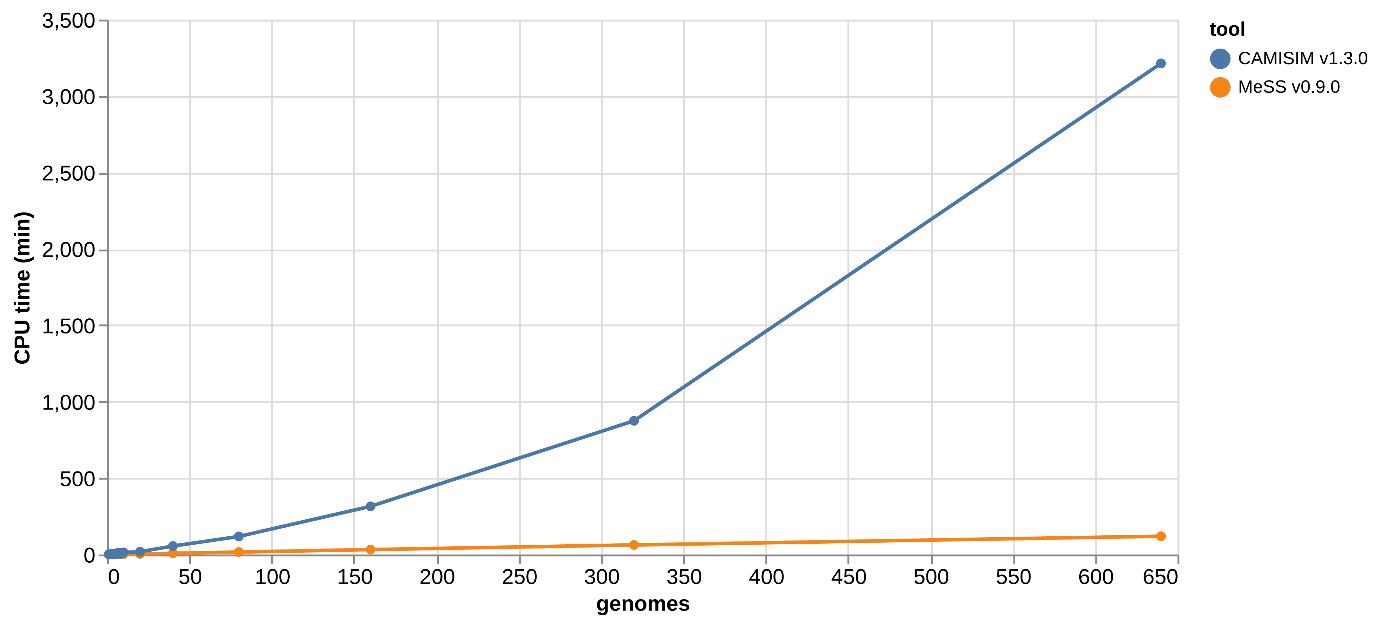

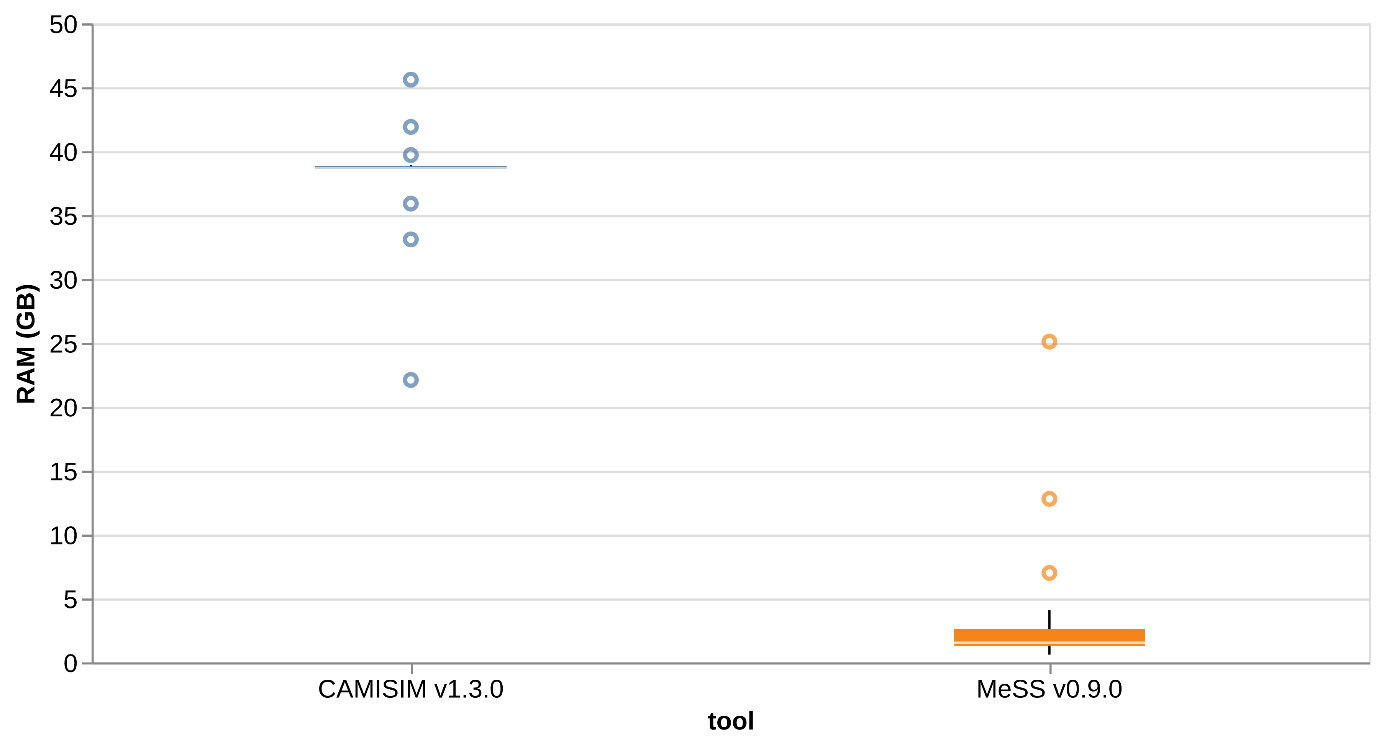


***Figure S2: Comparison of time complexity.*** *Process time (CPU time) in minutes according to the number of genomes used in metagenome simulation for CAMISIM (blue) and MeSS (orange).*

***Figure S3: Physical memory usage.*** *Boxplot comparing the physical memory usage in Gb between CAMISIM (blue) and MeSS (orange).*
